# Autochthonous *Plasmodium vivax* Infections, Florida, USA, 2023

**DOI:** 10.3201/eid3006.240336

**Published:** 2024-06

**Authors:** Azhar Muneer, Swamy R. Adapa, Suzane Silbert, Kelly Scanlan, Harold Vore, Andrew Cannons, Andrea M. Morrison, Danielle Stanek, Carina Blackmore, John H. Adams, Kami Kim, Rays H.Y. Jiang, Liwang Cui

**Affiliations:** University of South Florida Morsani College of Medicine, Tampa, Florida, USA (A. Muneer, K. Kim, L. Cui);; University of South Florida School of Public Health, Tampa (S.R. Adapa, J.H. Adams, K. Kim, R.H.Y. Jiang);; Tampa General Hospital, Tampa (S. Silbert, K. Kim);; Sarasota Memorial Hospital, Sarasota, Florida, USA (K. Scanlan, H. Vore);; Florida Department of Health Bureau of Public Health Laboratories, Tampa (A. Cannons);; Florida Department of Health, Tallahassee, Florida, USA (A.M. Morrison, D. Stanek, C. Blackmore)

**Keywords:** *Plasmodium vivax*, malaria, genome, origin, parasite introduction, parasites, vector-borne infections, local transmission, autochthonous, Florida, United States

## Abstract

During May–July 2023, a cluster of 7 patients at local hospitals in Florida, USA, received a diagnosis of *Plasmodium vivax* malaria. Whole-genome sequencing of the organism from 4 patients and phylogenetic analysis with worldwide representative *P. vivax* genomes indicated probable single parasite introduction from Central/South America.

Although commendable progress for combating malaria in endemic areas has been achieved and a dozen countries have been declared malaria-free since 2000 (*1*), increasing international travel has led to a rise in imported malaria cases, as well as sporadic cases of autochthonous malaria in non–malaria-endemic regions, especially those with competent vectors and favorable transmission conditions (*2*). In 2003 and 2023, two malaria outbreaks with local *Plasmodium vivax* transmission were reported in Florida (*3*,*4*). We examined the genomic characteristics, probable transmission dynamics, and likely origins of the 2023 *P. vivax* strains in Florida, demonstrating the role of genomic epidemiology in malaria control in non–malaria-endemic regions.

## The Study

**Figure 1:**
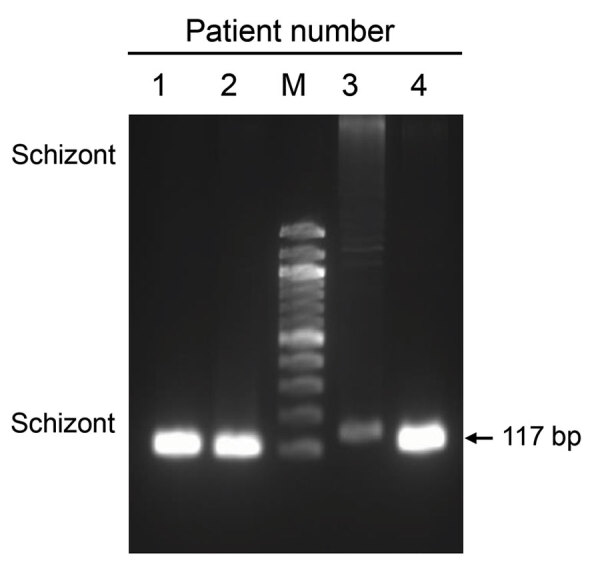
Identification of *Plasmodium vivax* infections in blood samples from malaria patients, Florida, USA, May–July 2023. Image shows 117-bp PCR products amplified from blood samples from 4 patients by using *P. vivax*–specific primers targeting the 18S rRNA gene. M, DNA ladder.

During May–July 2023, the Florida Department of Health received reports of a series of 7 cases of *P. vivax* malaria (*4*). The patients lacked risk factors for contracting malaria (e.g., recent histories of international travel, blood transfusion, or previous malaria), raising concerns for local mosquito transmission. All 7 patients were concentrated within a 4-mile radius, raising further concern about potential local transmission cycles. Pretreatment blood samples from 4 patients were collected on June 18, 20, 27, and July 12 (Appendix Figure 1). We used the anonymized remnant clinical samples collected for diagnosis purposes. Species-specific PCR of the samples (*5*) confirmed *P. vivax* infections (Figure 1). Three *Anopheles* mosquitoes collected in the affected region in June also tested positive for *P. vivax* (*4*).

To trace the origin of those *P. vivax* cases, we isolated DNA from 200 μL of whole blood from 4 patients by using a QIAamp DNA Blood Mini Kit (QIAGEN, https://www.qiagen.com). Recognizing the limited *P. vivax* DNA contaminated with overwhelming amounts of human DNA, we performed selective whole-genome amplification (sWGA) with *P. vivax*–specific primers (pvset1920) to enrich the *P. vivax* genome (*6*). Each 50-µL amplification reaction included 30 U of phi29 polymerase enzyme (New England Biolabs, https://www.neb.com), 1% bovine serum albumin, 2 mM of each of the 4 deoxynucleosides triphosphate, 3.5 µM of selective whole-genome amplification primers, and ≈70 ng of input DNA. Thermocycler conditions consisted of ramping down from 35°C to 30°C at a rate of 0.1°C per minute, 30°C for 16 hours, and 65°C for 10 minutes. The reaction product was purified with AMPure XP beads (Beckman-Coulter, https://www.beckmancoulter.com) and eluted in 50 µL Tris-EDTA buffer (pH 8). We obtained 10–12 μg of amplification product for each sample.

The sequencing libraries were prepared by using an Illumina DNA Prep Kit (https://www.illumina.com) and were pooled and sequenced for 100 million reads at 150-bp paired-end reads mode on the Illumina Nextseq 2000 platform. After trimming adapters and removing low-quality reads (*7*), we aligned high-quality reads to the *P. vivax* PvP01 reference genome by using the BWA-MEM (Burroughs-Wheeler Aligner–maximum exact matches) algorithm (*8*). We compressed, sorted, and indexed the alignment files by using SAMtools version 1.11 (*9*). For the 4 samples, we mapped 3%, 16%, 29%, and 55% of total reads to the PvP01 genome (Table), resulting in 7–124-fold and ≈72% coverage of the PvP01 genome. We deposited sequence data in the National Center for Biotechnology Information BioProject database (https://www.ncbi.nlm.nih.gov; BioProject no. PRJNA1093439).

**Table:** Whole-genome sequencing of *Plasmodium vivax* isolates from 4 malaria patients, Florida, USA, May–July 2023*

Patient no.	Sample collection date	Total reads	Reads mapped to PvP01, no. (%)	Mean PvP01 coverage, × (%)
1	Jun 18	39,350,480	1,163,557 (3)	7.0 (72.3)
2	Jun 20	41,417,476	6,725,684 (16)	40.2 (72.3)
3	Jun 27	35,086,122	10,243,361 (29)	72.6 (72.3)
4	Jul 12	37,493,726	20,596,607 (55)	124.2 (72.3)

*PvP01, reference genome for *Plasmodium vivax*.

We performed variant calling by using the GATK HaplotypeCaller (https://gatk.broadinstitute.org). To retain only high-confidence variants, we applied stringent quality filtering criteria to the VCF (variant call format) files. Comparison with the PvP01 sequence identified 97,180 high-quality single-nucleotide polymorphisms (SNPs), of which 38,799 were shared among all 4 Florida *P. vivax* isolates.

Next, we calculated the within-host fixation index for each patient sample, which was >0.95, indicating monoclonal infections. Of note, 57,656 (≈60%) SNPs were shared among samples 2, 3, and 4, indicating their close genetic relationships. The level of genetic variation was similar to the limited variation found in meiosis of single crosses (*10*), suggesting a limited local transmission event, likely seeded by a single introduction into Florida.

**Figure 2:**
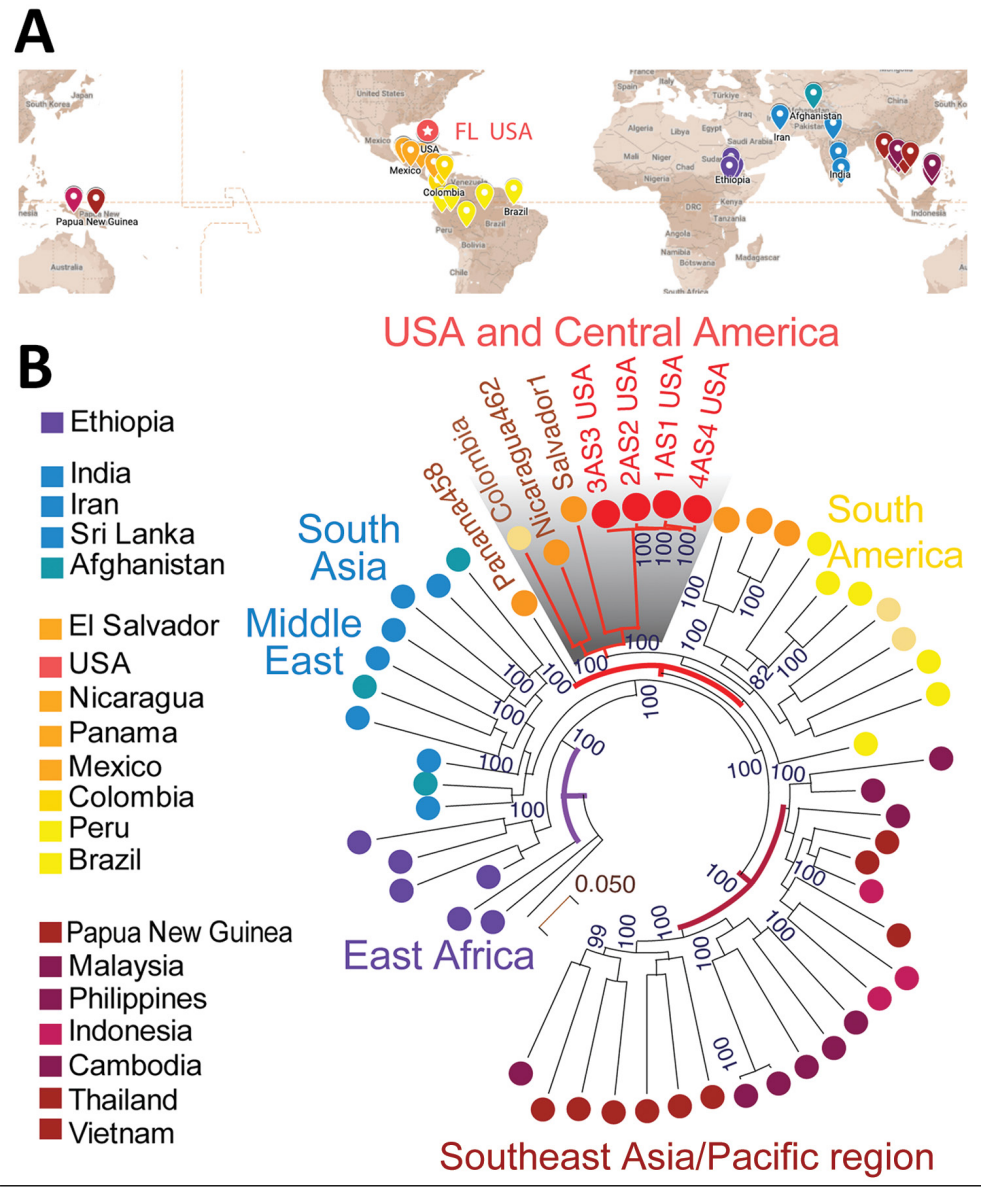
Phylogenetic analysis of *Plasmodium vivax* strains from blood samples from malaria patients, Florida, USA, May–July 2023, suggesting Central/South America origin. A) Geographic distribution of 53 high-quality global strains selected from >1,000 global *P. vivax* collections. B) Florida *P. vivax* strains clustering with Central/South America strains. The phylogenetic tree was constructed by using the maximum-likelihood method, and 1,000 bootstrap replications are shown next to the branches. The color coding of the geographic origin of the isolates matches the global map in panel A. The US and Central/South American cluster is shaded gray. The 4 Florida *P. vivax* strains are denoted as 1AS1, 2AS2, 3AS3, and 4AS4. Scale bar indicates nucleotide substitutions per site.

We constructed phylogeny between the Florida *P. vivax* isolates and 53 selected genomes from a global collection of 1,041 isolates (MalariaGen Pv4) (*11*). We chose a dataset consisting of high-quality *P. vivax* genome sequences from all continents where *P. vivax* is endemic (Figure 2, panel A). We constructed phylogenetic trees by using IQtree version 2.2.0 (*12*) and the general time-reversible model with ascertainment bias corrrection or the general time-reversible model with proportion of invariable sites and gamma-distributed rate heterogeneity. We evaluated both models with 1,000 ultrafast bootstrap approximation replicates and Shimodaira-Hasegawa–like approximate likelihood ratio test iterations. We assigned the final phylogenetic tree on the basis of bootstrap support values, ensuring robust statistical support for the inferred relationships. To ensure the reliability of the phylogenetic models, we allowed both models to converge; convergence was assessed at 300 iterations. We visualized and annotated the generated tree file by using Interactive Tree Of Life v.6 (*13*). Phylogenetic analysis with genome-wide SNPs revealed continental *P. vivax* population division (Figure 2, panel B) as previously reported (*11*). The *P. vivax* isolates from Florida, showing a pairwise genome distance of <0.01, formed a tight cluster (Appendix Figure 2), suggesting that they were probably the progeny of a single parasite isolate. Those isolates formed a highly supported Central/South America cluster (Figure 1). In sum, the timeline of the 7 cases (*3**,**4*) and our phylogenomic analysis support the interpretation of a single, limited introduction event from Central/South America into Florida. 

## Conclusions

Each year in the United States, ≈2,000 cases of imported malaria are reported to the Centers for Disease Control and Prevention (*14*). Although most cases are caused by *P. falciparum* infections, the 2003 and 2023 autochthonous outbreaks in Florida were caused by *P. vivax* (*3*,*4*). Similarly, local outbreaks from introduced *P. vivax* in Greece (*2*) and the Republic of Korea (*15*) illustrate the high potential for imported *P. vivax* to establish sustained transmission, which may pose a substantial challenge for subsequent elimination. Although the risk for autochthonous malaria in the United States remains low, the potential threat of imported *P. vivax* setting off and establishing local transmission in areas with competent vectors and conducive environments is a public health concern.

The last mosquito-vectored *P. vivax* malaria outbreak in the United States was in 2003 in Palm Beach County, Florida, and involved a cluster of 7 cases (*3*) before the *P. vivax* genome sequence was available. Although microsatellites were used to identify their genetic relatedness, the genomic tools that we used offer unparalleled resolution of the genetic relatedness of parasite isolates of the 2023 malaria outbreak in Florida. The minimal genetic variations among the isolates indicate a single transmission chain. Phylogenetic clustering with *P. vivax* strains from Central/South America implies the travel-related import of the index isolate from that region. Of note, a case of imported *P. vivax* malaria with symptom onset April 20, 2023, was reported from the same area as the 7 *P. vivax* cases (*3**,**4*). We are unable to determine if the April case initiated local transmission because no remaining specimen was available for analysis. 

In summary, our study underscores the usefulness and power of genomic tools in epidemiologic investigations. The established analytical pipeline will enable more streamlined and efficient genomic surveillance, provide health authorities with accurate information about the source of the infections, and enable communication of the risk to the public with solid scientific evidence. Given the rich genomic resources of worldwide *P. vivax* isolates, genomic surveillance will play a crucial role in tracking additional cases, detecting potential transmission chains, and identifying relapse. Although locally transmitted malaria in Florida has been successfully eliminated, ongoing surveillance, rapid responses, vigilance, and preparedness are still needed to prevent malaria reintroduction and local transmission.

Appendix Additional information for study of autochthonous *Plasmodium vivax* infections, Florida, USA, 2023.
