# Supplementary material for: Autochthonous Plasmodium vivax Infections, Florida, USA, 2023
Source: Emerg Infect Dis. 2024 Jun;30(6):1214–7. doi: 10.3201/eid3006.240336 (PMC11138989; doi:10.3201/eid3006.240336)
Supplement: Appendix — Additional information for study of autochthonous Plasmodium vivax infections, Florida, USA, 2023. [file 24-0336-Techapp-s1.pdf]

Article DOI: https://doi.org/10.3201/eid3006.240336

EID cannot ensure accessibility for supplementary materials supplied by authors. Readers who have difficulty accessing supplementary content should contact the authors for assistance.

Autochthonous *Plasmodium vivax* Infections, Florida, USA, 2023

Appendix

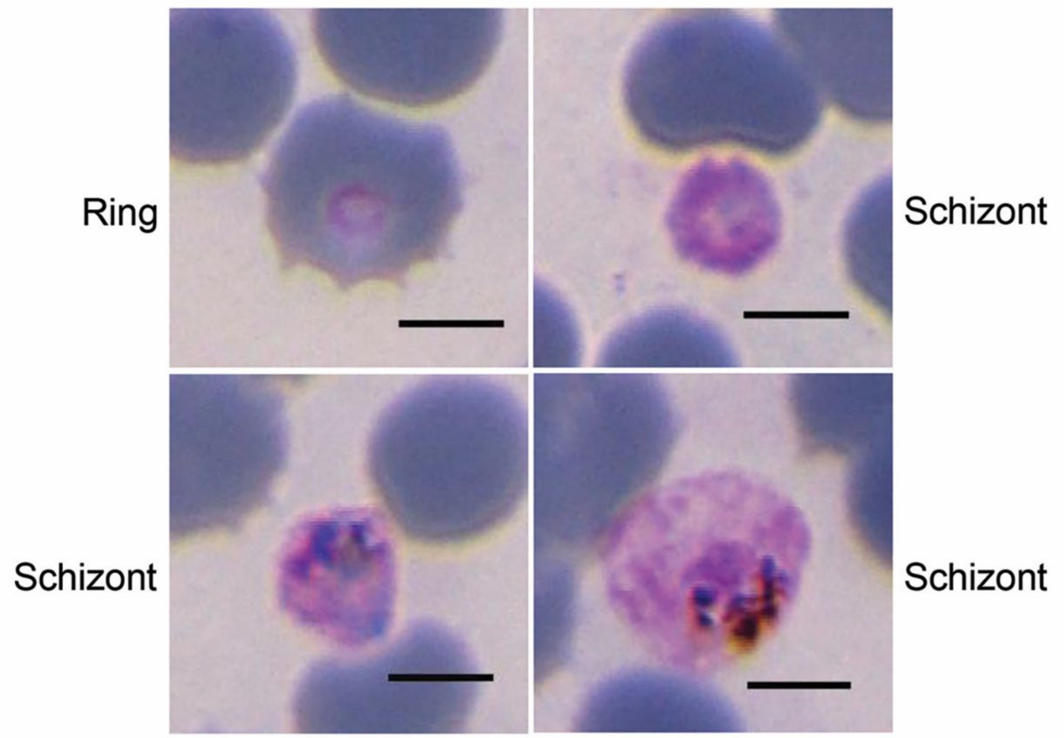

Appendix Figure 1. Identification of *Plasmodium vivax* infections in blood samples from malaria patients, Florida, USA, May–July 2023. Microscopic images of Giemsa-stained thin blood smear from 1 patient, showing developmental stages of the *P. vivax* parasite. Scale bars indicate 5 μm.

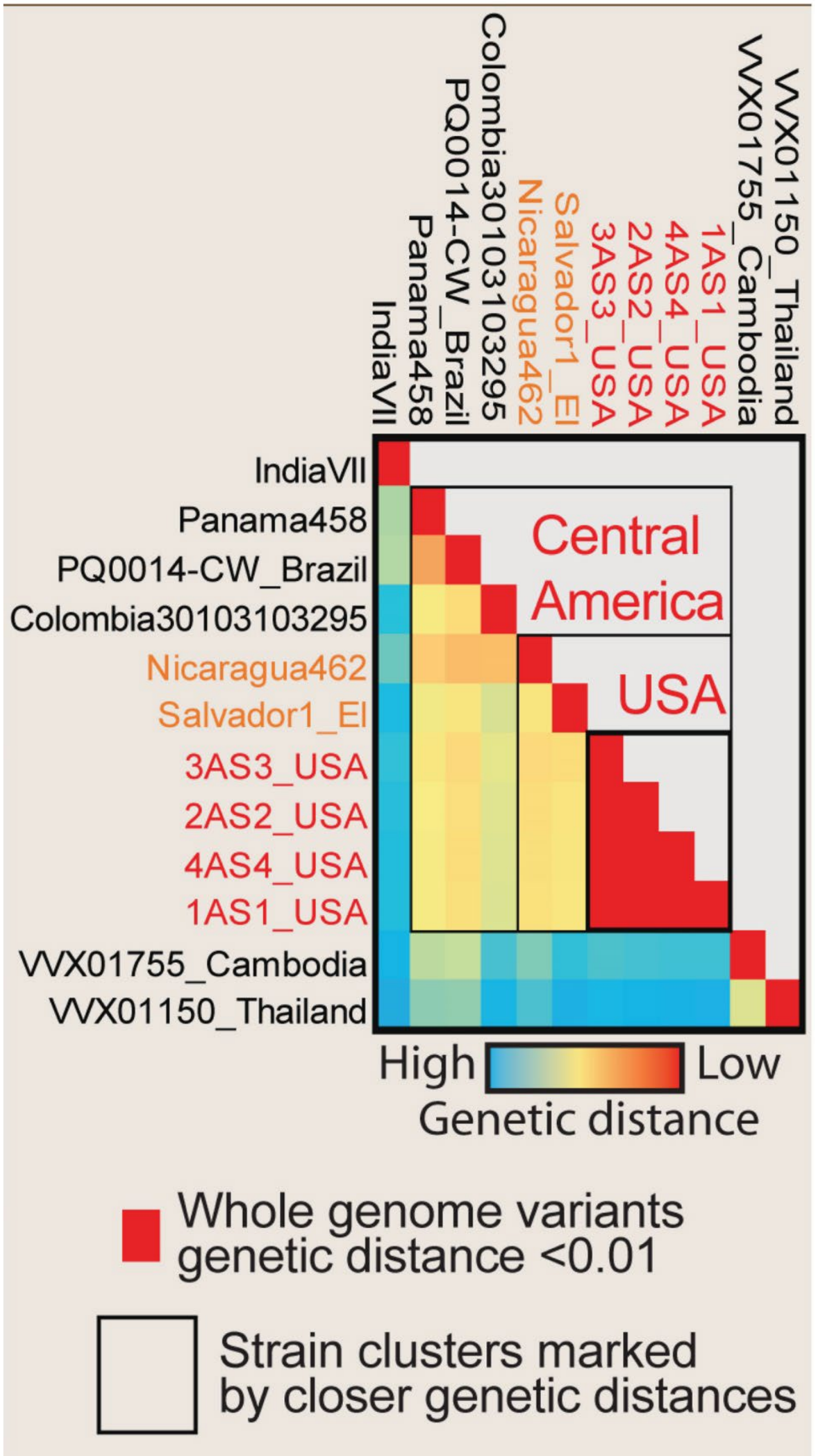

Appendix Figure 2. Phylogenetic analysis of *Plasmodium vivax* strains from blood samples from malaria patients, Florida, USA, May–July 2023, suggesting Central/South American origin. The distance matrix shows that US strains are closely related. The distances of the number of substitutions per site are shown. Strains in red font, United States; orange font, Central/South America; black font, other regions.
